# A prismatic view of protein phosphorylation in health and disease

**DOI:** 10.3389/fgene.2015.00131

**Published:** 2015-04-07

**Authors:** Allegra Via, Andreas Zanzoni

**Affiliations:** ^1^Department of Physics, Sapienza University of RomeRome, Italy; ^2^Technological Advances for Genomics and Clinics (TAGC), UMR_S1090, INSERMMarseille, France; ^3^Technological Advances for Genomics and Clinics (TAGC), UMR_S1090, Aix Marseille UniversitéMarseille, France

**Keywords:** protein phosphorylation, disease, evolution, cell signaling, systems biology, bioinformatics, phosphorylation networks

The paramount relevance of protein phosphorylation in health and disease motivated us to gather several contributions from experts working in this area in order to portray the recent developments in the field. Our effort and the effort of 54 authors with their 12 contributions gave rise to this Research Topic, which represents a valuable forum where phosphorylation is discussed from different angles, including bioinformatics approaches and experimental methods that are currently used to decipher the complex mechanisms underlying this bewitching post-translational modification (PTM). The articles collected in this Research Topic illustrate very diverse aspects of phosphorylation, such as its biological effects and induced structural changes, the role of kinases and phosphatases as therapeutic targets, the use of phosphorylation profiles as biomarkers, how phosphorylation dys-regulation may cause disease, and more.

Nishi et al. (2014), in their extensive review of the representative studies on the biological effects of phosphorylation, show that a general mechanism of regulation by phosphorylation does not exist. Indeed, phosphorylation may serve as recognition/binding site or trigger allosteric effects inducing local structural changes, which may propagate into larger structure rearrangements. Some nice examples of the biological consequences of protein phosphorylation in physiological and disease states are described in two articles of this Research Topic.

The Hsp27 protein (coded by the HSPB1 gene) is a chaperone that is aberrantly expressed in many types of tumors and represents a promising drug target (Acunzo et al., 2014). Phosphorylation of serine residues affects the oligomerization state of Hsp27, favoring the recruitment of different client proteins involved in distinct cellular functions (Katsogiannou et al., 2014a). Katsogiannou et al. (2014b) suggest that better understanding Hsp27 phosphorylation dynamics in cancer may help improve existing and/or develop new therapies.

Amata et al. (2014) report the interesting case of the role of phosphorylation events in the Unique domain of Src family kinases (SFKs). This domain, an intrinsically disordered region little conserved across the family and linking the kinase to its membrane-anchoring domain, is stubbed with phosphosites involved in multilevel regulation of SFKs, including, among others, anchoring to the lipid membrane.

These examples hint at the high complexity of the cellular networks regulated by phosphorylation. Kinases and phosphatases can be also regulated by phosphorylation and most of signal transduction pathways involve cascades of phosphorylation and de-phosphorylation events. This scenario should give the reader a glimpse of the consequences on entire pathways of the abolition or stimulation of phosphorylation caused, for instance, by mutational events. In this context, the development of experimental and computational methods aimed at understanding the mechanisms of specificity and functioning of kinase and phosphatase repertoires is becoming increasingly relevant.

A comprehensive review (Newman et al., 2014) presents a broad picture on well established and emerging experimental methods that are providing new insights on the organization and regulation of phosphorylation networks. Thanks to these approaches, thousands of phosphorylation events have been identified in distinct cellular conditions. Importantly, these technological advances are stimulating the development of computational models to gain a systems-level understanding of phosphorylation maps (e.g., Linding et al., 2007; Song et al., 2012; Newman et al., 2013).

A full comprehension of such maps requires the elucidation of the molecular determinants of substrate recognition. The review of Palmeri and collaborators summarizes the current knowledge on kinase-substrate specificity (Palmeri et al., 2014), arguing that only the combination of different sources of contextual information will provide new insights into the molecular determinants of kinase specificity and improve the performance of kinase-substrate interaction prediction tools.

The understanding of kinase specificity is also particularly relevant for the identification, design and development of compounds modulating the kinase activity. In this context, reliable computational methods for kinase/inhibitor inference and analyses would play a crucial role. As pointed out by Ferrè et al. (2014), such methods are being successfully developed and applied to the whole kinome thanks to the increasing amount of homogeneous data provided by high-throughput profiling studies.

Most inhibition screenings focus on compounds able to bind the kinase catalytic site. However, on one hand, targeting the catalytic site lacks specificity and, on the other, the modulation of kinase activity can be achieved also through alternative routes. An illustrative example, is presented by Gonfloni in a perspective article reviewing the current knowledge of c-Abl regulation by allosteric compounds, such as GNF-2 (Gonfloni, 2014). The author proposes that the use of current and novel allosteric activators will elucidate the role of c-Abl regulation in cancer and neurodegenerative disorder signaling pathways.

This example suggests that rational drug design would tremendously benefit from a better comprehension of kinase conformational changes and flexibility. Whilst experimental techniques able to accurately describe such phenomena are still under development, computational methods for the study of kinase conformational transitions—especially Atomistic Molecular Dynamics (MD)—demonstrated to be of great help. In their minireview, D'Abramo et al. (2014) highlight that, since kinase conformational switches from inactive to active states occur on long time-scales, enhanced sampling techniques and brute-force approaches are being developed and successfully applied to rational drug design in a growing number of cases.

Despite phosphatases are key players in phosphorylation networks, they caught less attention than kinases as drug targets and the identification of phosphatase physiological substrates is still an open challenge (Brautigan, 2013).

In order to tackle this problem, Sacco and colleagues (Sacco et al., 2014), in their original research article, present computational an integrative approach combining functional siRNA information (Sacco et al., 2012), interaction discovery experiments and network analyses to identify phosphatase substrates and potential scaffolds proteins that could mediate substrate recognition. Their strategy was able to recover known as well as novel phosphatase substrates/scaffolds, some of which were further validated.

The growing knowledge of phosphatases and their substrates is expanding the interest of the scientific community in these enzymes as potential therapeutic targets in order to modulate dys-regulated phosphorylation levels of disease proteins. One example is provided by Taymans and Baekelandt (2014) who review phosphorylation dys-regulation in the three main proteins linked to Parkinson Disease: alpha-synuclein, Leucine-rich repeat kinase type 2 (LRRK2), and microtubule associate protein tau (tau). They analyze the feasibility of targeting their phosphatases as a potential therapy for Parkinsonism.

Together with the study of kinases and phosphatases, the analysis of phosphorylation profiles is also important in developing therapies or biomarkers for phosphorylation-related diseases.

In an original research article, Robertson and colleagues (Robertson et al., 2014) describe an integrative approach to study the honeybee (*Apis mellifera*) kinome with the aim of detecting differences in phosphorylation profiles between bees with differential susceptibility to Varroa mite infestation at different developmental stages. They found that many peptides are differentially phosphorylated between the two phenotypes and bioinformatics analyses showed clear differences between resistant and susceptible phenotypes.

The evolution of PTMs and phosphorylation in particular, is another key aspect of the story. In a minireview, Landry et al. (2014) present recent evidences suggesting that clusters of sites rather than individual sites represent functional units evolving under stabilizing selection. Nevertheless, more experimental studies are needed to test whether the stabilizing selection model applies to phosphosite clusters.

With the multifaceted description of protein phosphorylation presented in this Research Topic, we hope we provided the readers with a taste of the huge complexity of phosphorylation networks and pathways and convinced them that a deeper knowledge of the interplay between kinases, phosphatases and their substrates is essential in the quest for new disease biomarkers and novel therapeutic targets.

Finally, we would like to thank all the authors for their pivotal contribution to this Research Topic, as well as the 25 peers that acted as review editors and the Frontiers in Genetics Editorial Office staff members for their support.

## Conflict of interest statement

The authors declare that the research was conducted in the absence of any commercial or financial relationships that could be construed as a potential conflict of interest.
